# Inverse Modulation of Neuronal K_v_12.1 and K_v_11.1 Channels by 4-Aminopyridine and NS1643

**DOI:** 10.3389/fnmol.2018.00011

**Published:** 2018-01-30

**Authors:** Marlen Dierich, Saskia Evers, Bettina U. Wilke, Michael G. Leitner

**Affiliations:** ^1^Department of Neurophysiology, Institute of Physiology and Pathophysiology, Philipps University of Marburg, Marburg, Germany; ^2^Division of Physiology, Department of Physiology and Medical Physics, Innsbruck Medical University, Innsbruck, Austria

**Keywords:** K_v_10, K_v_11, K_v_12, HERG, mode shift, voltage-dependent potentiation, 4-aminopyridine, NS1643

## Abstract

The three members of the *ether-à-go-go-gene*-like (Elk; K_v_12.1-K_v_12.3) family of voltage-gated K^+^ channels are predominantly expressed in neurons, but only little information is available on their physiological relevance. It was shown that K_v_12.2 channels modulate excitability of hippocampal neurons, but no native current could be attributed to K_v_12.1 and K_v_12.3 subunits yet. This may appear somewhat surprising, given high expression of their mRNA transcripts in several brain areas. Native K_v_12 currents may have been overlooked so far due to limited knowledge on their biophysical properties and lack of specific pharmacology. Except for K_v_12.2, appropriate genetically modified mouse models have not been described; therefore, identification of K_v_12-mediated currents in native cell types must rely on characterization of unique properties of the channels. We focused on recombinant human K_v_12.1 to identify distinct properties of these channels. We found that K_v_12.1 channels exhibited significant mode shift of activation, i.e., stabilization of the voltage sensor domain in a “relaxed” open state after prolonged channel activation. This mode shift manifested by a slowing of deactivation and, most prominently, a significant shift of voltage dependence to hyperpolarized potentials. In contrast to related K_v_11.1, mode shift was not sensitive to extracellular Na^+^, which allowed for discrimination between these isoforms. Sensitivity of K_v_12.1 and K_v_11.1 to the broad-spectrum K^+^ antagonist 4-aminopyridine was similar. However, 4-AP strongly activated K_v_12.1 channels, but it was an inhibitor of K_v_11 channels. Interestingly, the agonist of K_v_11 channels NS1643 also differentially modulated the activity of these channels, i.e., NS1643 activated K_v_11.1, but strongly inhibited K_v_12.1 channels. Thus, these closely related channels are distinguished by inverse pharmacological profiles. In summary, we identified unique biophysical and pharmacological properties of K_v_12.1 channels and established straightforward experimental protocols to characterize K_v_12.1-mediated currents. Identification of currents in native cell types with mode shift that are activated through 4-AP and inhibited by NS1643 can provide strong evidence for contribution of K_v_12.1 to whole cell currents.

## Introduction

The ether-à-go-go (Eag) superfamily of voltage-gated K^+^ channels comprises three evolutionary conserved families that share high sequence homology: Ether-à-go-go (Eag; K_v_10), ether-à-go-go-related-gene (Erg; K_v_11) and ether-à-go-go-gene-like (Elk; K_v_12) channels (Bauer and Schwarz, 2001). The best-studied member, K_v_11.1 (the human isoform is referred to as HERG channel) mediates rapidly activating K^+^ current I_Kr_ in cardiac myocytes determining heart action potential duration (Sanguinetti et al., 1995). Accordingly, loss of K_v_11.1 channel function through mutations or drug treatment causes cardiac arrhythmia and sudden death in humans (Curran et al., 1995; Sanguinetti et al., 1995; Trudeau et al., 1995). K_v_11 channels also mediate important K^+^ currents in neurons of the auditory brainstem (Hardman and Forsythe, 2009), the olfactory bulb (Hirdes et al., 2009) and the midbrain (Ji et al., 2012). K_v_10.1 channels regulate cell cycle progression and proliferation (Sanchez et al., 2016; Urrego et al., 2016), and are frequently overexpressed in human cancers with poor prognosis (Pardo and Stuhmer, 2014). K_v_10.1 channel mutations cause developmental disorders and epilepsy (Kortum et al., 2015; Simons et al., 2015).

In contrast to K_v_10 and K_v_11 channels, only little information on physiological relevance is available for the three members of the K_v_12 family that are expressed predominantly in neurons (Engeland et al., 1998; Shi et al., 1998; Miyake et al., 1999; Trudeau et al., 1999; Saganich et al., 2001; Zou et al., 2003). K_v_12.2 channels regulate excitability in pyramidal neurons of hippocampus in mice (Zhang et al., 2010), but no native current component could be attributed to K_v_12.1 and K_v_12.3 subunits despite expression of their mRNA transcripts in several brain areas (Shi et al., 1998; Miyake et al., 1999; Saganich et al., 2001; Zou et al., 2003). We consider that K_v_12.1/K_v_12.3-mediated currents in neurons were overlooked so far due to insufficient knowledge on biophysical properties and lack of specific pharmacological tools.

**FIGURE 1:**
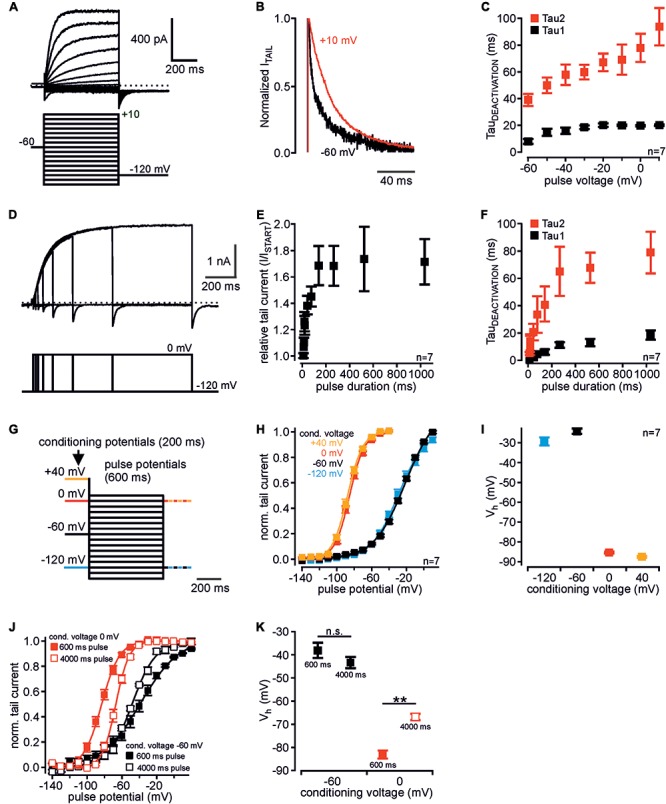
Voltage-dependent mode shift of human K_v_12.1 channels. **(A)** Representative patch clamp recording of a CHO cell transiently transfected with human K_v_12.1 channels measured with the indicated voltage protocol. **(B)** Representative tail currents elicited at –120 mV after activating pulses of –60 mV (black) and +10 mV (red). Currents were normalized to maximum amplitude for visualization of deactivation kinetics (currents from recording shown in **A**). **(C)** Tail current deactivation was best described by double exponential kinetics: Tau1 and tau2 were derived from double-exponential fits to the decaying phase of tail currents (voltage protocols as in **A**). Deactivation slowed down with channel activation at more depolarized potentials. **(D–F)** When K_v_12.1 channels were activated at 0 mV for different time intervals, **(E)** tail current amplitudes increased and **(F)** deactivation slowed-down with pre-pulse duration. Tail current increase and slowing of deactivation saturated at a pulse duration of about 200 ms and 400–500 ms, respectively (**D** shows a representative recording). **(G–I)** Analysis of voltage dependence of recombinant K_v_12.1 channels. **(G)** Voltage protocols consisted of a 200 ms conditioning potential step to –120 mV (blue), –60 mV (black), 0 mV (red), or +40 mV (orange), followed by 600 ms activating pulse potentials from –140 to +10 mV (10 mV increments). In these experiments, tail currents were elicited either at –120 mV or at 0 mV (for representative recordings see Supplementary Figures 1A,C). **(H)** Summary of voltage dependence of human K_v_12.1 channels derived from Boltzmann fits to individual recordings as shown in Supplementary Figures 1A,C (solid line represents a Boltzmann fit to averaged data). Depolarized conditioning potentials of +40 and 0 mV induced a large shift of voltage dependence to hyperpolarized potentials. **(I)** shows mean *V*_h_ of channel activation in dependence of conditioning potentials (derived from fits shown in **H**). **(J,K)** Upon extension of the activating pulses from 600 ms (filled black squares) to 4000 ms (open black squares), voltage dependence of recombinant K_v_12.1 channels was not significantly changed when measured after a conditioning pulse of –60 mV. However, extension of the activating pulse to 4000 ms significantly shifted voltage dependence of K_v_12.1 channels to depolarized potentials after conditioning voltage of 0 mV (*P* ≤ 0.01; open red squares). For data shown in **(J)** eight independent recordings were averaged (each cell measured with all four voltage protocols). There was no significant difference between corresponding data in **(J,K)** and **(H,I)** (conditioning potential –60 mV, 600 ms).

Recently, it was shown that K_v_12.1 channels exhibit mode shift of activation (also termed pre-pulse facilitation or voltage-dependent potentiation) (Li et al., 2015; Dai and Zagotta, 2017). Mode shift denotes time-dependent stabilization of the voltage sensor domain in a “relaxed” open state after prolonged channel activation through depolarized (conditioning) membrane potentials (Bezanilla et al., 1982; Villalba-Galea et al., 2008). It manifests by slowing of deactivation and a shift of voltage dependence to hyperpolarized potentials (Li et al., 2015; Dai and Zagotta, 2017). Accordingly, when measured with routine voltage clamp protocols (e.g., holding potentials of -60 mV) human K_v_12.1 channels mediate “conventional” K^+^ currents that activate similar to many other K_v_ channels with voltages at half-maximal activation of around -30 mV (c.f. **Figure 1**) (Li et al., 2015). These currents could easily go unnoticed in cell types expressing different endogenous K^+^ currents. Taking into account their mode shift, appropriate voltage protocols (e.g., depolarized holding potentials) may uncover K_v_12-mediated currents. However, it remains to be explored whether mode shift can be detected in cells expressing various K^+^ currents and whether it may be employed to identify native K_v_12.1 channels. Mode shift is not an exclusive feature of K_v_12.1 channels, but has been demonstrated also for voltage-gated Na^+^ channels (Bezanilla et al., 1982), HCN (Mannikko et al., 2005; Bruening-Wright and Larsson, 2007), *Shaker* (Olcese et al., 1997; Tilegenova et al., 2017), and K_v_11.1 (Erg1) channels (Piper et al., 2003; Tan et al., 2012; Goodchild et al., 2015). Nevertheless, it may constitute a prominent hallmark to distinguish K_v_12 channels from other K^+^ current components in native tissue.

Here we describe biophysical and pharmacological properties of K_v_12.1 channels and demonstrate straightforward experimental protocols that may be employed to identify K_v_12 currents in neurons. We show, that these properties allowed detection of K_v_12.1-mediated currents in cells expressing a variety of different K^+^ channels. Our findings may be utilized to identify physiological roles of K_v_12.1 channels.

## Results

### Voltage-Dependent Mode Shift of Human K_v_12.1 Channels

Mode shift of human K_v_12.1 channels was recently demonstrated (Li et al., 2015), but a detailed characterization is currently not available. In order to identify exclusive properties of human K_v_12.1 channels, we thus set out with detailed biophysical analysis of these channels in an overexpression system. In CHO cells, activation of K_v_12.1 channels through depolarizing voltage steps produced robust outwardly rectifying currents (**Figure 1A**) (Zou et al., 2003; Li et al., 2015; Dai and Zagotta, 2017). Channel deactivation at hyperpolarized potentials was best described by double exponential kinetics, and deactivation slowed down with more depolarized pre-potentials (**Figures 1B,C**). When we activated K_v_12.1 channels at 0 mV for different time intervals, tail current amplitudes increased (**Figures 1D,E**) and deactivation slowed down with the duration of the pre-pulse (**Figure 1F**). Tail current increase and slowing of deactivation saturated at a pulse duration of about 200 ms and 400–500 ms, respectively.

**FIGURE 2:**
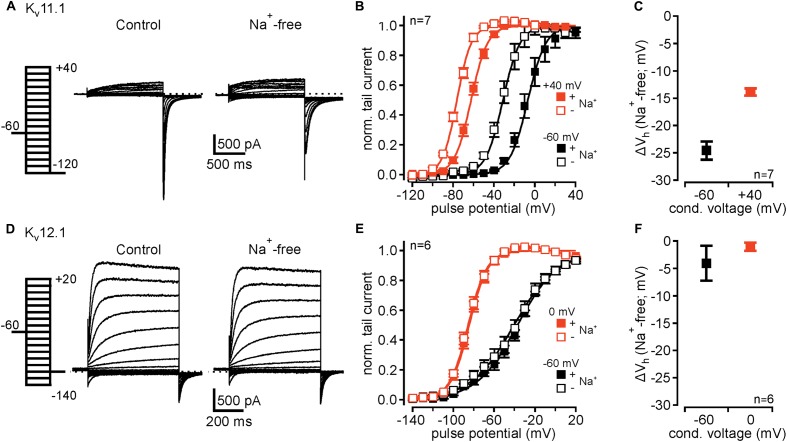
K_v_12.1 are not sensitive to extracellular Na^+^. **(A)** Representative recordings from a CHO cell transiently transfected with K_v_11.1 under control conditions (left) and after removal of external Na^+^ (right). **(B)** K_v_11.1 channels exhibit mode shift of activation, as previously reported. After conditioning potential of +40 mV voltage dependence of K_v_11.1 channels shifted to hyperpolarized potentials, compared to conditional potential of –60 mV [solid lines represent a Boltzmann fit to averaged data; from recordings as shown in **(A)**]. Upon removal of extracellular Na^+^, voltage dependence of K_v_11.1 shifted to hyperpolarized potentials after conditioning potentials of –60 and +40 mV. **(C)** Shows averaged shifts of *V*_h_ (Δ*V*_h_) for conditional potentials of –60 and +40 mV after removal of extracellular Na^+^. **(D–F)** Recombinant K_v_12.1 channels are insensitive to extracellular Na^+^. **(D)** Representative recordings of K_v_12.1-mediated currents under control conditions (left) and in absence of extracellular Na^+^ (right). **(E)** Summary of voltage dependence of K_v_12.1 channels derived from experiments presented in **(D)**. Note that neither current amplitudes nor voltage dependence of K_v_12.1 channels were affected by removal of extracellular Na^+^. **(F)** shows that removal of extracellular Na^+^ did not shift *V*_h_ (Δ*V*_h_) of activation of K_v_12.1 channels. In these experiments, Na^+^ in the extracellular solution was replaced by NMDG without changing extracellular K^+^ concentration.

We then analyzed voltage dependence of human K_v_12.1 channels with voltage protocols established previously to study mode shift of related K_v_11.1 (Tan et al., 2012). We applied depolarizing holding potentials (conditioning potentials; 200 ms) before a series of activating voltage steps (pulse potentials from -140 mV to +10 mV; 600 ms) (**Figure 1G** and Supplementary Figures 1A–C). To minimize time intervals at hyperpolarized potentials that may counteract mode shift (c.f. Villalba-Galea, 2017), we at start recorded tail currents at correspondingly depolarized potentials (**Figures 1G–I**). In these experiments, amplitudes of K_v_12.1-mediated outward currents were similar irrespective of conditioning potentials. This indicated that comparable steady-state channel activation was reached with all voltage protocols (Supplementary Figures 1A–C). After a conditioning potential of -60 mV, voltage dependence of K_v_12.1 channels also did not change relevantly upon extension of the activating steps to 4000 ms (**Figures 1J,K**). This additionally demonstrated that steady-state activation of K_v_12.1 channels was already reached by activating pulses as short as 600 ms. For 600 ms activating pulses, half-maximal voltages of activation (*V*_h_) were -29.3 ± 2.3 mV and -24.1 ± 1.6 mV for negative conditioning potentials of -120 mV or -60 mV, respectively (*n* = 7; 600 ms activating pulses; **Figures 1H,I**). When cells were held at depolarized conditioning pulses of 0 mV or +40 mV, *V*_h_ was -85.3 ± 0.9 mV and -87.6 ± 0.8 mV, respectively (**Figures 1H,I**; 600 ms activating pulses). In these experiments, slope factors derived from Boltzmann fits to the recordings changed from -15.7 ± 0.7 mV (conditioning pulse of -120 mV) and -16.4 ± 0.9 mV (-60 mV) to -8.6 ± 0.1 mV (0 mV) and -8.9 ± 0.6 (+40 mV) (*n* = 7; Supplementary Figure 1D; 600 ms activating pulses). Accordingly, depolarizing conditioning potentials induced a large shift of voltage dependence by about -60 mV, and the full shift occurred across a potential range of 60 mV (between holding potentials of -60 and 0 mV). In analogous experiments, the same conditioning voltages shifted *V*_h_ of related K_v_11.1 channels from -6.8 ± 4.4 mV (conditioning voltage -60 mV) to -62.4 ± 1.5 (conditioning voltage +40 mV; *n* = 7; **Figures 2A,B**), consistent with a previous report (Tan et al., 2012).

We next tested whether mode shift was sensitive to the employed voltage protocol. Therefore, we again applied 200 ms depolarized conditioning pulses, but this time we extended the activating pulses to 4000 ms (**Figures 1J,K**). When in these experiments cells were held at conditioning pulses of -60 and 0 mV, *V*_h_ was -43.3 ± 2.5 mV and -66.8 ± 1.5 mV, respectively (**Figures 1J,K**). Thus, mode shift of K_v_12.1 channels was readily induced also when activating the channels for 4000 ms. However, the extent of mode shift was significantly reduced in these experiments compared to experiments with 600 ms activating pulses (**Figure 1K**; *P* ≥ 0.01), i.e., increased time intervals at hyperpolarized holding potentials during these protocols presumably counteracted development of mode shift. Similarly, the extent of mode shift was reduced when we activated channels for 600 ms after conditioning pulses of -60 and 0 mV, but recorded tail currents at hyperpolarized holding potentials (-120 mV; Supplementary Figures 1E,F), i.e., we introduced additional hyperpolarizing potentials after every activating pulse. Thus, also in these experiments hyperpolarizing holding potentials between the conditioning pulses reduced the expression of mode shift.

Taken together, human K_v_12.1 channels exhibited significant voltage-dependent mode shift that in response to depolarized holding potentials manifested by slowed channel deactivation and by a large hyperpolarizing shift of voltage dependence. This mode shift of K_v_12.1 channels can be induced robustly employing different voltage protocols, but the extent of mode shift significantly varies with duration of hyperpolarized holding potentials.

### In Contrast to K_v_11.1, K_v_12.1 Channels Are Insensitive to Extracellular Na^+^

Inhibition of K_v_11 channels by extracellular Na^+^ is well established (Numaguchi et al., 2000; Sturm et al., 2005) and a hallmark used to identify K_v_11-mediated currents in neurons (e.g., Hardman and Forsythe, 2009). In control experiments, replacement of extracellular Na^+^ with NMDG without altering extracellular K^+^ concentration slightly increased K_v_11.1-mediated outward currents (**Figure 2A**), as previously reported (Numaguchi et al., 2000). Upon removal of extracellular Na^+^, activation voltage range of K_v_11.1 channels conditioned at -60 and +40 mV shifted to hyperpolarized potentials by -24.4 ± 1.5 mV and -13.9 ± 0.7 mV, respectively (*n* = 7; **Figures 2B,C**). Accordingly, overall mode shift of voltage dependence was attenuated from -55.7 ± 4.4 mV under control conditions to -45.1 ± 3.5 mV in absence of extracellular Na^+^ (*n* = 7; *P* ≤ 0.05). We then tested whether related K_v_12.1 channels also exhibited such Na^+^ sensitivity. We found that neither current amplitudes nor voltage dependence of K_v_12.1 channels were affected by removal of extracellular Na^+^ (**Figures 2D–F**). Consequently, the extent of mode shift was also insensitive to changes of the Na^+^ concentration. To conclude, despite the high similarity in mode shift behavior, the absence of Na^+^ sensitivity in K_v_12.1 channels distinguishes K_v_11.1-mediated currents from K_v_12.1.

### K_v_12.1 Channels Are Not Sensitive to K_v_ Channel Blockers E-4031, XE991, and TEA

We then evaluated whether K_v_12.1 channels were sensitive to channel inhibitors that are widely used to attribute neuronal K^+^ currents to particular channel families. At concentrations generally applied to inhibit established target channels, human K_v_12.1 channels were insensitive to both E-4031 (20 μM), a specific inhibitor of K_v_11 channels (Supplementary Figures 2A,B) (Trudeau et al., 1995), and XE991 (10 μM), a specific antagonist of neuronal K_v_7 (KCNQ) channels (Supplementary Figures 2D,E) (Wang et al., 1998). K_v_12.1-mediated currents were also insensitive to E-4031 and XE991 concentrations up to 100 μM (Supplementary Figures 2C,F). K_v_12.1 channels also were not affected by the broad-spectrum K^+^ channel inhibitor tetraethylammonium (TEA) at a concentration that completely inhibited K_v_7.2 channels (5 mM; Supplementary Figures 2G,H) (Hadley et al., 2000). However, K_v_12.1 channels were slightly inhibited by 50 and 100 mM TEA (*I*_50mM TEA_/*I*_Start_ = 0.90 ± 0.01; *I*_100mM TEA_/*I*_Start_ = 0.83 ± 0.01; *n* = 9; Supplementary Figure 2I). Of note, low sensitivity of K_v_12.1 channels to E-4031 and TEA has been shown in an early report (Shi et al., 1998). In summary, E-4031, XE991 and TEA cannot be used to inhibit K_v_12.1 channels in native tissue, but may be used to isolate K_v_12.1 channel activity through inhibition of other K^+^ channels.

### The Broad-Spectrum K^+^ Channel Antagonist 4-Aminopyridine (4-AP) Activates K_v_12.1

**FIGURE 3:**
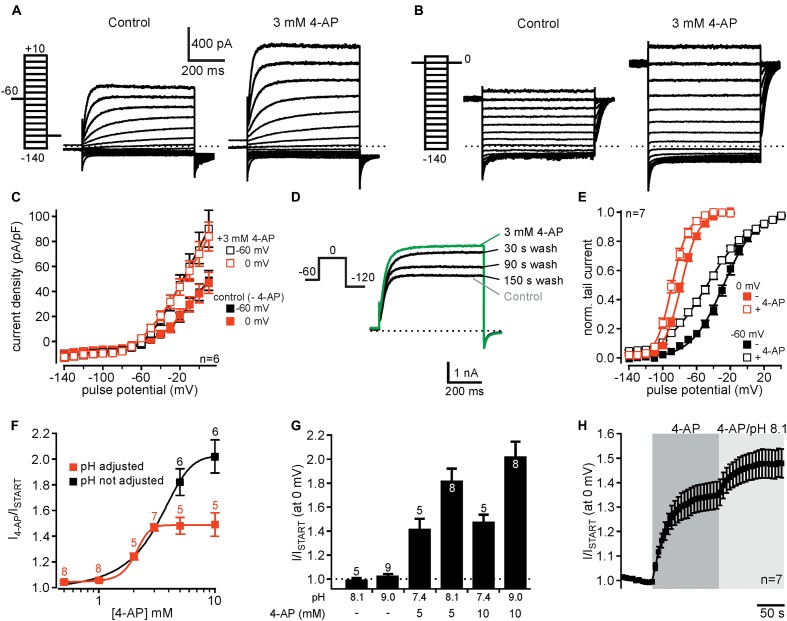
4-AP potentiates currents through human K_v_12.1 channels. **(A,B)** Representative recordings from a CHO cell transiently transfected with K_v_12.1 before (left) and after extracellular application of 3 mM 4-AP (right) after conditioning pulses of **(A)** –60 mV and **(B)** 0 mV. **(C)** 4-AP-dependent activation of K_v_12.1-mediated steady-state outward currents was independent on employed voltage protocols (summary of recordings as presented in **A,B**). 4-AP potentiated currents at all holding potentials positive to –60 mV. **(D)** 4-AP-dependent potentiation of K_v_12.1 currents was reversible, as current amplitudes returned to control levels within 2–3 min after removal of the substance [shown are representative recordings under control conditions (gray), after application of 3 mM 4-AP (green) and at different time points (30, 90, and 150 s) after washout of 4-AP]. **(E)** 3 mM 4-AP shifted the voltage dependence of human K_v_12.1 channels to hyperpolarized potentials (open squares) [the panel shows Boltzmann fits to averaged data; data obtained from recordings as shown in **(A,B)**]. **(F)** 4-AP activated K_v_12.1 channels in a dose-dependent manner. When pH of the extracellular solution was adjusted to 7.4 at higher 4-AP concentrations, the EC_50_ was about 2.1 mM and the Hill coefficient was about 0.8 (red trace). When pH of the solutions was not adjusted, the EC_50_ was about 3.1 and Hill coefficient was 0.9 (black trace). Parameters were derived from fits of averaged data to a Hill equation described in Section “Materials and Methods” (solid line represents fit; note that addition of 4-AP to 5 and 10 mM 4-AP increased the pH of the solution to 8.1 and 9.0, respectively). **(G)** Increasing the pH of the extracellular solution from 7.4 to 8.1 or 9.0 without addition of 4-AP did not increase K_v_12.1-mediated steady-state currents. In contrast, application of 5 mM or 10 mM 4-AP in extracellular solution without adjusting pH activated K_v_12.1 channels even more strongly than when pH of the solution was adjusted to pH 7.4. **(H)** Application of 3 mM 4-AP that does not change pH of the extracellular solution strongly potentiated K_v_12.1-mediated currents. Current potentiation through 3 mM 4-AP was further increased when the pH of the extracellular solution was increased to pH 8.1.

We then tested whether human K_v_12.1 channels were sensitive to 4-AP, another established antagonist of several K_v_ families (Gutman et al., 2005). Surprisingly, 4-AP at a concentration well in the range often used to isolate neuronal K^+^ currents (3 mM; e.g., Marcotti et al., 2003) increased K_v_12.1-mediated steady-state and tail currents within seconds (**Figures 3A–C**). Current potentiation was the same for conditioning pulses of -60 and 0 mV, and 4-AP potentiated currents at all holding potentials positive to -60 mV (**Figure 3C**). 4-AP-dependent current increase was reversible within 2–3 min after washout of the drug (**Figure 3D**). 4-AP (3 mM) not only potentiated K_v_12.1-mediated currents, but also significantly shifted the voltage dependence of K_v_12.1 channels to hyperpolarized potentials (**Figure 3E**). Compared to control recordings measured before application of the substance, 3 mM 4-AP shifted *V*_h_ by -19.7 ± 1.7 mV (*n* = 7; *P* ≤ 0.001) and by -10.4 ± 1.5 mV (*n* = 7; *P* ≤ 0.001) after condition voltage pulses of -60 and 0 mV, respectively. Thus, the shift of *V*_h_ was significantly more pronounced for currents conditioned at -60 mV (*P* ≤ 0.01; c.f. **Figure 3E**).

We then analyzed the dose-response relationship of 4-AP action on K_v_12.1 channels. As 5 and 10 mM 4-AP increased pH of the solution to about 8.1 and 9.0, respectively, we adjusted pH of these solutions to 7.4 after addition of 4-AP. Of note, 3 mM 4-AP or lower 4-AP concentrations did not alter the pH of the solution relevantly and thus no adjustment of pH was necessary. Under these experimental conditions, 4-AP potentiated K_v_12.1 currents in a concentration-dependent manner with an EC_50_ of about 2.1 and a Hill coefficient of about 0.8 (**Figure 3F**, *red trace*). In contrast, K_v_11.1 channels were inhibited by 4-AP with an IC_50_ of approximately 2.6 mM and a Hill coefficient of about 0.7 (Supplementary Figure 3), consistent with previous reports (e.g., Ridley et al., 2003). Thus, despite inverse effects of 4-AP on K_v_12.1 and K_v_11.1, the sensitivity of both channels to 4-AP was quite similar. When we applied 5 mM or 10 mM 4-AP without adjusting pH, we found that these concentrations activated K_v_12.1 channel even stronger than at physiological pH (**Figures 3F,G**). Without adjusting pH at higher concentrations, the EC_50_ was about 3.1 and the Hill coefficient was 0.9 (**Figure 3F**, *black trace*). As in line with a previous study (Kazmierczak et al., 2013) increasing pH of the extracellular solution from 7.4 to 8.1 or 9.0 without addition of 4-AP did not potentiate K_v_12.1-mediated steady-state currents (**Figure 3G**), these data suggested that 4-AP activated K_v_12.1 channels more efficiently at more alkaline pH. Indeed, increasing pH of the extracellular solution containing 3 mM 4-AP to 8.1 further increased K_v_12.1-mediated steady-state outward currents (**Figure 3H**).

### K_v_12.1 Channels Are Also Activated by Isomeric Aminopyridines

**FIGURE 4:**
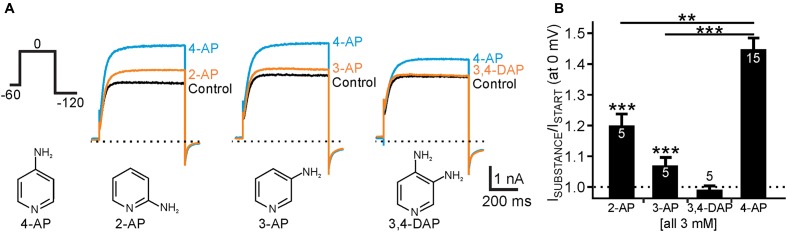
Activation of human K_v_12.1 channels by isomeric aminopyridines. **(A)** Representative whole cell currents through human K_v_12.1 channels elicited by voltage steps to 0 mV (voltage protocol as indicated). The upper panel shows effects of 2-AP (left), 3-AP (middle), or 3,4-DAP (right) on human K_v_12.1 channels. 4-AP was applied at the end of every recording for comparison of current potentiation (concentration of all substances was 3 mM). The lower panel displays chemical structures of 4-AP, 2-AP, 3-AP, and 3,4-DAP. **(B)** Summary of recordings as presented in **A** (steady-state outward currents at 0 mV were analyzed at the end of the activating pulse). At the same concentration (all 3 mM), 2-AP and 3-AP significantly potentiated K_v_12.1-mediated currents, albeit to a lower extent than 4-AP (^∗∗^*P* ≤ 0.01; ^∗∗∗^*P* ≤ 0.001).

Several other aminopyridines have been shown to inhibit voltage-gated K^+^ channels, albeit antagonistic efficiency of these substances was lower than that of 4-AP (Robertson and Nelson, 1994; Sedehizadeh et al., 2012; Strupp et al., 2017). We thus wondered whether we could identify isomeric aminopyridines that activated K_v_12.1, at best without affecting other K^+^ channels. We found that 2-aminopyridine (2-AP) and 3-aminopyridine (3-AP) potentiated K_v_12.1-mediated currents at a concentration close to the EC_50_ of 4-AP (3 mM) by about 20% (*P* ≤ 0.001) and 7% (*P* ≤ 0.001), respectively (**Figure 4**). At the same concentration 3,4-diaminopyridine (3,4-DAP; 3 mM) was ineffective. Hence, 2-AP (*P* ≤ 0.01) and 3-AP (*P* ≤ 0.001) activated K_v_12.1 channels significantly less than 4-AP mirroring efficacy of inhibition of other K^+^ channels by these substances (Robertson and Nelson, 1994; Sedehizadeh et al., 2012; Strupp et al., 2017).

### NS1643, an Activator of K_v_11 Channels, Inhibits K_v_12.1 Channels

**FIGURE 5:**
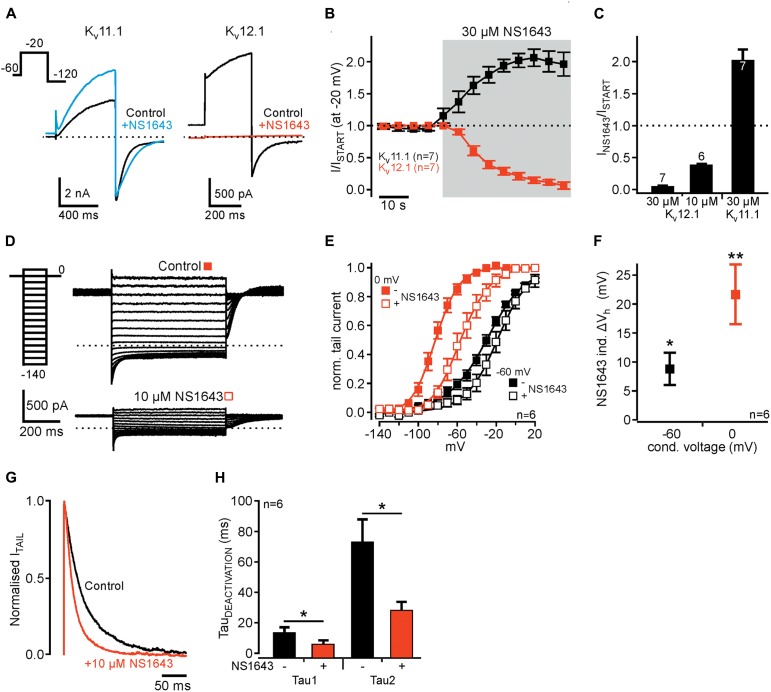
Human K_v_12.1 channels are inhibited by NS1643. **(A)** Representative recordings of recombinant K_v_11.1 (left) and K_v_12.1 (right) channels before (black) and after (colored) application of 30 μM NS1643 (voltage protocol as indicated). **(B)** Averaged time course of the amplitudes of outward currents mediated by K_v_11.1 (black) and K_v_12.1 (red) channels upon application of 30 μM NS1643. NS1643 activated K_v_11.1 channels [as previously reported; (Casis et al., 2006; Hansen et al., 2006)], but completely inhibited K_v_12.1 channels (data were derived from recordings as in **A**). **(C)** Summarized effects of NS1643 on K_v_11.1 and K_v_12.1 channels. 10 μM NS1643 did not completely inhibit K_v_12.1 channels allowing for further analysis of remaining currents. The time course of K_v_12.1 current inhibition by 10 μM NS1643 is shown in Supplementary Figure 4. **(D–F)** Voltage dependence of K_v_12.1 channels changes through application of 10 μM NS1643. **(D)** Representative recordings of K_v_12.1 channels after conditioning potential of 0 mV before (top) and after (bottom) application of 10 μM NS1643 (for clarity only these recordings are shown). **(E)** 10 μM NS1643 significantly shifted voltage dependence of K_v_12.1 channels to depolarized potentials (solid lines represent Boltzmann fits to averaged data). **(F)** Shows averaged shifts of *V*_h_ (Δ*V*_h_) induced by 10 μM NS1643 for currents conditioned at –60 and 0 mV. **(G,H)** NS1643 significantly accelerated K_v_12.1 channel deactivation. **(G)** Representative normalized tail currents elicited at –120 mV after activation at –20 mV before (control, black) and after (red) application of 10 μM NS1643. **(H)** Shows averaged time constants of deactivation before and after application of NS1643 (10 μM) (^∗^*P* ≤ 0.05; ^∗∗^*P* ≤ 0.01).

We then turned to NS1643, a partial agonist of the K_v_11 channel family (Casis et al., 2006). As shown earlier (c.f. Hansen et al., 2006), NS1643 (30 μM) slowed channel deactivation and potentiated K_v_11.1-mediated outward currents (**Figures 5A–C**). In contrast, the same concentration of NS1643 (30 μM) completely inhibited K_v_12.1 channels with a time constant of 16.8 ± 2.6 s (*n* = 7; **Figures 5A–C**). When we applied only 10 μM NS1643, a concentration close to the reported EC_50_ of NS1643 for activation of K_v_11.1 channels (Casis et al., 2006), K_v_12.1-mediated currents were reduced to 37.1 ± 4.1% of initial current amplitudes. Inhibition of currents by 10 μM was much slower than when 30 μM NS1643 was applied (*n* = 6; compare **Figure 5B** and Supplementary Figure 4). We then analyzed voltage dependence of residual K_v_12.1 currents in the presence of 10 μM NS1643 (**Figures 5D–F**): Application of NS1643 (10 μM) shifted *V*_h_ by +8.8 ± 2.9 mV (*n* = 6; *P* ≤ 0.05) and by +21.7 ± 5.2 mV (*n* = 6; *P* ≤ 0.01) after condition voltage pulses of -60 and 0 mV, respectively (**Figures 5D–F**). At the same time, slope factors changed by +8.0 ± 1.5 mV (conditioning pulse of -60 mV; *n* = 6; *P* ≤ 0.05) and -1.8 ± 0.9 mV (conditioning pulse 0 mV; *n* = 6; *P* ≤ 0.01). Furthermore, NS1643 (10 μM) significantly accelerated deactivation of K_v_12.1 channels at hyperpolarized potentials (**Figures 5G,H**; *P* ≤ 0.05; *n* = 6).

### Identification of K_v_12.1 Currents in Cells Expressing Different K^+^ Currents

**FIGURE 6:**
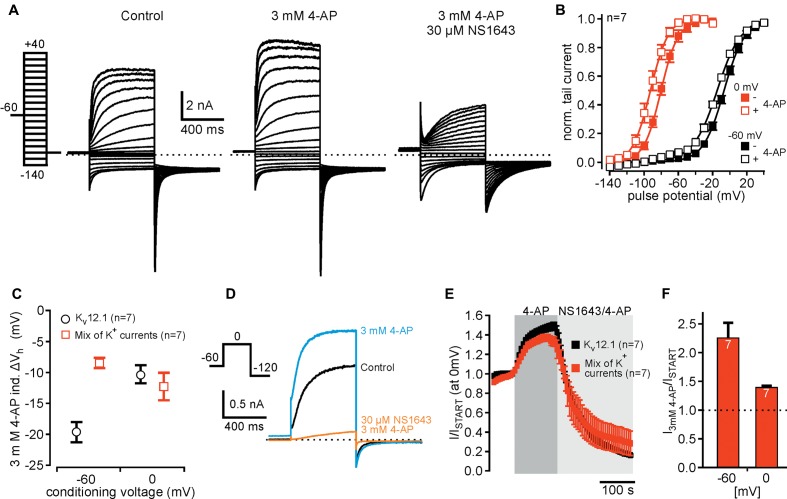
Detection of K_v_12.1 channel activity in cells expressing various K^+^ currents. **(A)** Representative recordings of a CHO cell expressing different K^+^ channel subunits (K_v_7.2, K_v_7.3, K_v_11.1 and Kir2.1) together with K_v_12.1 under control conditions (left), upon application of 3 mM 4-AP (middle) and of 4-AP together with 30 μM NS1643 (right) (voltage protocol as indicated). **(B)** Voltage dependence of whole cell currents in cells expressing the mix of K^+^ channel subunits. *V*_h_ of whole cell currents was –6.7 ± 1.3 mV (black filled squares) after conditioning potential of –60 mV. When cells were held for 200 ms at a conditioning potential of 0 mV before the activation pulses, *V*_h_ of whole cell currents was –80.0 ± 2.1 mV (red filled squares). Application of 3 mM 4-AP shifted voltage dependence to hyperpolarized potentials for both conditioning potentials (open squares; graph displays Boltzmann fits to averaged data as solid lines). **(C)** 4-AP (3 mM)-induced shift of *V*_h_ to hyperpolarized potentials shown for cells expressing the mix of K^+^ channels (red squares) in comparison to cells expressing only K_v_12.1 (black circles). The 4-AP-induced shift of voltage dependence after conditioning voltage of –60 mV was smaller than in cells expressing only K_v_12.1 channels. Data for K_v_12.1 alone were derived from experiments also shown in **Figure 3E**. **(D,E)** Summary of time course and current amplitudes in cells expressing a mix of K^+^ channels during during application of 4-AP and NS1643 (together with 4-AP). **(D)** Representative current traces recorded in cells expressing a mix of K^+^ channels before (black), during application of 3 mM 4-AP (blue) and during application of 3 mM 4-AP together with 30 μM NS1643 (orange). **(E)** Averaged time course of current amplitudes upon application of 3 mM 4-AP and 30 μM NS1643 for CHO cells expressing K_v_12.1 channels alone (black) and the mix of K^+^ channels (red) (data obtained from recordings as presented in **D**). **(F)** Summarized 4-AP-induced potentiation of steady-state outward currents for cells expressing K_v_12.1 channels together with a mix of neuronal K^+^ channels. Data were derived from recordings as presented in **(D)**, and currents elicited at –60 mV and at 0 mV were analyzed.

Our results suggested that voltage-clamp protocols designed to detect mode shift in combination with pharmacology using 4-AP or NS1643 should provide a robust approach for isolation of K_v_12.1-mediated currents in native cell types. As proof of principle, we sought to isolate K_v_12.1 channel activity in cells expressing different K^+^ channels (**Figure 6**). To this end, we co-expressed K_v_12.1 channels together with K_v_11.1 and typical neuronal K^+^ channels (K_v_7.2, K_v_7.3 and Kir2.1). In these experiments, CHO cells were transiently transfected with equal amounts of plasmid DNA encoding the channel subunits (see section “Materials and Methods” for details). We first analyzed whether we could isolate Kir2.1- and K_v_7-mediated currents in those cells. In all cells tested, we found large inward currents at hyperpolarized potentials and XE991-sensitive outward currents at depolarized potentials demonstrating expression of functional Kir2.1 and K_v_7 channels, respectively (Supplementary Figure 5). As measure for abundance of K_v_11.1 and K_v_12.1 channels, we then tested whether we could detect mode shift of voltage dependence in the mix of K^+^ currents. As determined from whole cell currents, *V*_h_ was -6.7 ± 1.3 mV and -80.0 ± 2.1 mV after conditioning potentials of -60 and 0 mV, respectively (*n* = 7; **Figures 6A–C**). Thus, mode shift of channels expressed in these cells (K_v_11.1 and K_v_12.1 channels) was readily detectable even among a complement of different voltage-dependent K^+^ channels. We next attempted to isolate K_v_12.1-mediated currents among the mixture of K^+^ channel by making use of the pharmacological profile established above. 4-AP (3 mM) shifted the voltage dependence of whole cell currents by -8.5 ± 0.9 mV (*n* = 7; *P* ≤ 0.001) and by -12.3 ± 2.3 mV (*n* = 7; *P* ≤ 0.05) to hyperpolarized potentials after conditioning voltages of -60 and 0 mV, respectively (**Figures 6B,C**). Thus, although being less pronounced than for K_v_12.1 channels alone (**Figure 6C**), the 4-AP-induced shift of voltage dependence was readily detectable in the mixed K^+^ current situation. At the same time, 4-AP (3 mM) potentiated whole cell currents elicited at -60 and 0 mV to about 210% and 140% of baseline amplitudes, respectively (**Figures 6A,D–F**). Finally, we applied 30 μM NS1643 (on top of 4-AP) to the same cells and found that the substance inhibited outward currents and inward tail currents (**Figures 6A,D,E**). NS1643 also slowed deactivation kinetics of remaining currents (c.f. **Figure 6A**) indicating that NS1643-sensitive and functional K_v_11.1 channels were expressed in these cells.

Taken together, using abovementioned experimental protocols we could unequivocally identify properties of K_v_12.1 channels (mode shift, 4-AP and NS1643 sensitivity) among a mixture of K^+^ currents. These results therefore suggest that the same protocols may be used to assign K^+^ current components to K_v_12.1 channels in complex (native) cellular/neuronal settings.

## Discussion

The three members of the *ether-à-go-go*-like channel family of voltage-gated K^+^ channels (K_v_12.1-K_v_12.3) are predominantly expressed in neurons, as shown by mRNA transcripts in several brain areas including cerebral cortex and hippocampus (Miyake et al., 1999; Trudeau et al., 1999; Saganich et al., 2001; Zou et al., 2003), as well as in sympathetic ganglia (Shi et al., 1998). However, neuronal current components and their physiological relevance have been resolved for K_v_12.2 subunits exclusively: as demonstrated by application of a selective inhibitor, K_v_12.2 activity controls resting membrane potential and spontaneous firing of pyramidal neurons in hippocampus (Zhang et al., 2010). Also, in K_v_12.2 knock-out mice it was shown that K_v_12.2 channels regulate hippocampal excitability (Zhang et al., 2010) and may be important for processing of spatial working memory (Miyake et al., 2009). However, it is not known whether K_v_12 channels mediate relevant currents in any other physiological system.

Here, we focused on recombinant human K_v_12.1 to identify distinct biophysical and pharmacological properties allowing for attribution of native currents to these channels.

### Human K_v_12.1 Channels Exhibit Mode Shift of Activation

Mode shift of activation was recently demonstrated for K_v_12.1 channel isoforms of humans and zebrafish heterologously expressed in *Xenopus laevis* oocytes (Li et al., 2015; Dai and Zagotta, 2017). However, mode shift of these channels has not been characterized in mammalian cells in detail yet. We found that mode shift of human K_v_12.1 was readily induced by depolarized holding potentials between -60 and 0 mV and that it manifested by slowed channel deactivation and a striking shift of voltage dependence to hyperpolarized potentials. Deceleration of deactivation is generally considered a biophysical hallmark of mode shift, as channels undergo additional (time-consuming) transitions from this “relaxed” (metastable) open state into deactivation (Bezanilla et al., 1982; Villalba-Galea et al., 2008; Corbin-Leftwich et al., 2016; Villalba-Galea, 2017). Similar to K_v_11.1 (Tan et al., 2012) and zebrafish K_v_12.1 (Dai and Zagotta, 2017), mode shift of human K_v_12.1 channels manifested by a striking -60 mV shift of voltage dependence. Thus, mode shift-dependent hyperpolarizing shifts of voltage dependence were qualitatively and quantitatively comparable for these three ion channels. Of note, mode shift of K_v_12.1 channels was readily induced using different voltage protocols, albeit the extent of the hyperpolarizing shift of activation potentials varied considerably with duration of hyperpolarized voltage steps. This demonstrated high sensitivity of K_v_12.1 channels to the holding potential, but also highlighted that voltage dependence of native K_v_12.1 currents might also vary with the used voltage protocols. In contrast to zebrafish K_v_12.1 (Dai and Zagotta, 2017), activation of human K_v_12.1 channels did not exhibit prominent double exponential kinetics that might indicate transition into a more stable open conformation. However, human K_v_11.1 channels that beyond doubt exhibit mode shift do not display such kinetics neither (Tan et al., 2012; Goodchild et al., 2015). This indicates that either kinetics were masked by channel inactivation, or transition was too fast in the human channel isoforms.

Time course of transition into the relaxed state varies considerably between channels requiring depolarization for minutes in Na_v_ (Bezanilla et al., 1982), seconds in *Shaker* (Olcese et al., 1997) and some hundreds of milliseconds in K_v_11.1 channels (Piper et al., 2003). For human K_v_12.1, we found that also some hundreds of milliseconds of conditioning depolarization was sufficient for significant alterations of voltage dependence and kinetics. Thus, time course of development of K_v_12.1 mode shift was well in the range of that published for K_v_11.1 channels (Piper et al., 2003). As K_v_12.1 channels are highly sensitive to changes of the holding potential, voltage dependence of the channels strongly depends on the employed voltage protocols. In fact, such protocol differences could account for considerable variations in voltage dependence of K_v_12.1 channels reported in different studies (*V*_h_ close to 0 mV in Shi et al., 1998;*V*_h_ of about -60 mV in Zou et al., 2003).

### K_v_12.1 Channels Are Activated by 4-AP, an Established K_v_ Channel Inhibitor

4-AP is a rather selective blocker of voltage-gated K^+^ channels: At micromolar concentrations, it reversibly inhibits activity of K_v_1 and K_v_3 family members, but at higher concentrations (in the millimolar range) 4-AP also blocks other K_v_ channels (e.g., K_v_11) (Gutman et al., 2005; Alexander et al., 2015). Thus, it was somewhat surprising to find that 4-AP activates K_v_12.1 channels. As an early study mentioned potentiation of K_v_12.3 through 4-AP without, however, showing any recordings (named rELK1; Engeland et al., 1998), such 4-AP sensitivity may constitute a general feature of the K_v_12 family.

Earlier, it was shown that currents through K_v_2.1 channels that are normally inhibited by 4-AP were potentiated by the substance, but only when K_v_6.4 subunits were co-expressed (Stas et al., 2015). As K_v_2.1 and K_v_6.4 co-assemble into functional channels (reviewed in Bocksteins, 2016), this suggested that K_v_6.4 largely determined altered 4-AP sensitivity of the resulting heteromeric channels. 4-AP suppressed closed state inactivation of K_v_2.1/K_v_6.4 resulting in exclusive current potentiation of currents through those heteromers (Stas et al., 2015). In contrast, K_v_12.1 channels did not inactivate in our experiments, and thus 4-AP probably does not potentiate K_v_12.1-mediated currents through a similar mechanism. As 4-AP, in contrast to K_v_2.1/K_v_6.4 heteromers, also modulated voltage dependence of K_v_12.1, actions of 4-AP are probably even more complex for these channels. Interestingly, a recent study demonstrated 4-AP-dependent activation of K_v_7.4 channels (Khammy et al., 2017) indicating that 4-AP-dependent activation of voltage-gated K^+^ channels may constitute a more frequent phenomenon than expected. However, further work is needed to elucidate whether other members of the K_v_ channel superfamily also exhibit this special 4-AP sensitivity.

Yet, we do not know whether 4-AP directly activates K_v_12.1 channels or whether an auxiliary subunit endogenously expressed in CHO cells confers 4-AP activation to K_v_12.1. Unfortunately, at present nothing is known about physiologically relevant interaction partners of K_v_12.1 channels. As K_v_ channels (with K_v_2.1 as exception) normally do not form functional channels with members of other K_v_ families, heteromerization of K_v_12.1 channels with another pore forming α subunit apart from K_v_12.2 or K_v_12.3 is quite unlikely (Zou et al., 2003). Accordingly, a mechanism as shown for K_v_2.1 is rather implausible, and it is hard to imagine how a non-pore-forming auxiliary subunit could reverse 4-AP sensitivity from inhibition to activation. Furthermore, any endogenously expressed auxiliary subunit would need to be expressed at high abundance to saturate overexpressed K_v_12.1 channels. Hence, a straightforward model proposes direct activation of K_v_12.1 channels through 4-AP. However, we want to point-out that we cannot exclude indirect actions on the channels at the moment.

### NS1643, a “Specific” Activator of K_v_11 Channels, Inhibits K_v_12.1

NS1643 is a well-characterized partial agonist of K_v_11 channels that slows deactivation, increases tail-current amplitude, and shifts voltage dependence of activation to hyperpolarized potentials and voltage dependence of C-type inactivation to depolarized potentials (Casis et al., 2006; Hansen et al., 2006). At the same time, NS1643 exhibits weak antagonistic effects on K_v_11 channels as evident by an attenuation of drug-induced current increase at higher concentrations (Casis et al., 2006; Schuster et al., 2011). K_v_11.3 channels are even inhibited by higher concentrations of NS1643. For K_v_12.1 channels, NS1643 accelerated deactivation, inhibited outward and tail currents and shifted voltage dependence of activation to depolarized potentials (c.f. **Figure 5**). Thus, NS1643 affected K_v_11 and K_v_12.1 channels exactly in opposite directions. Similar to K_v_11.3 channels, NS1643 increased slope factor of voltage dependence of K_v_12.1 channels, even though already at lower concentrations. This suggests that NS1643 similarly affects K_v_11 and K_v_12.1 channels, but antagonistic effects might dominate over activation for K_v_12.1. In line, the concentration range of NS1643 effects was similar for these channel isoforms (Casis et al., 2006). However, so far we cannot tell whether NS1643 also exhibits weak agonistic effects on K_v_12.1 channels or whether NS1643 binds to homologous regions in K_v_11 and K_v_12.1 channels.

### Conclusion and Outlook

As pharmacological tools and appropriate mouse models are currently missing, identification of native K_v_12.1-mediated currents critically depends on identification of unique biophysical and pharmacological properties. Indeed, native currents were successfully attributed to related K_v_10 and K_v_11 channels by exploiting their unique activation kinetics (c.f. Cole-Moore shift; e.g., Meyer and Heinemann, 1998) or exclusive Na^+^ sensitivity and pharmacology (Hirdes et al., 2005, 2009; Hardman and Forsythe, 2009), respectively. Here, we present distinctive pharmacological properties, and straightforward experimental protocols that may be employed to isolate K_v_12.1 channel activity in native tissue. As mode shift readily manifested in cells expressing various neuronal K^+^ currents, this experimental protocol may be used to demonstrate expression of channels with mode shift in native cell types. In neurons expressing various K^+^ current components, such changes of voltage dependence may be easier to detect than associated changes of deactivation kinetics. Such experiments may not provide definite proof for K_v_12 channel expression. However, expression of mode shift may be employed together with expression analyses, Na^+^ sensitivity, activation through 4-AP and inhibition by NS1643 to narrow down (or exclude) contribution of K_v_12.1 channels to whole cell currents. Identification of the combination of these properties would provide strong evidence for expression and thus potential physiological relevance of K_v_12.1 channels.

## Materials and Methods

### Cell Culture and Transfection

Chinese hamster ovary (CHO) dhFR^-^ cells were maintained as previously reported (Leitner et al., 2016). Cells were kept in MEM Alpha Medium supplemented with 10% fetal calf serum (FCS) and 1% penicillin/streptomycin (pen/strep) (all Invitrogen GmbH, Darmstadt, Germany) in a humidified atmosphere at 5% CO_2_ and 37°C. Transient transfection of cells was done with jetPEI transfection reagent (Polyplus Transfection, Illkirch, France). All experiments were performed 24–48 h after transfection at room temperature (22–25°C). The expression vectors used were: K_v_7.2-pBK-CMV (gene: human KCNQ2; UniProt accession number: O43526), K_v_7.3-pBK-CMV (human KCNQ3; O43525), K_v_7.4-pBK-CMV (human KCNQ4; P56696) K_v_11.1 (Erg1)-pcDNA3.1 (rat KCNH2; O08962), K_v_12.1(Elk1)-pcDNA3.1-IRESeGFP (human KCNH8; Q96L42), Kir2.1-pBK-CMV (human KCNJ2; P63252), and pEGFP-C1 (Addgene, Teddington, United Kingdom). For recordings presented in **Figure 6** and Supplementary Figure 5, CHO cells were transiently transfected with identical amounts of plasmids encoding K_v_7.2, K_v_7.3, K_v_11.1, K_v_12.1, and Kir2.1 (0.6 μg of plasmid DNA for every subunit).

### Electrophysiological Recordings

Electrophysiological recordings were performed in the whole cell configuration with an Axopatch 200B amplifier (Molecular Devices, Union City, CA, United States) or an HEKA EPC10 USB patch clamp amplifier HEKA (Elektronik, Lambrecht, Germany) in voltage-clamp mode (Leitner et al., 2011). All recordings were low-pass filtered at 2 kHz and sampled at 5 kHz. Currents were elicited by voltage protocols indicated in the figures. Dashed lines in representative recordings highlight zero current. Borosilicate glass patch pipettes (Sutter Instrument Company, Novato, CA, United States) were used with an open pipette resistance of 2–3 MΩ after back-filling with intracellular solution. Liquid junction potentials were not compensated (approximately -4 mV). Series resistance (*R*_s_) typically was below 6 MΩ and compensated throughout the recordings (80–90%). Whole cell currents are presented normalized to the cell capacitance (current density; pA/pF) or as normalized to baseline current amplitude (*I*/*I*_Start_). Extracellular solutions for most experiments contained (mM) 144 NaCl, 5.8 KCl, 1.3 CaCl_2_, 0.9 MgCl_2_, 0.7 NaH_2_PO_4_, 10 HEPES and 5.6 D-glucose, pH 7.4 (with NaOH), 305–310 mOsm/kg. In some experiments, NaCl in the extracellular solution was substituted by *N*-methyl-D-glucamine (NMDG; Sigma–Aldrich) (c.f. **Figure 2**). The standard intracellular solution contained (mM) 135 KCl, 2.41 CaCl_2_ (100 nM free Ca^2+^), 3.5 MgCl_2_, 5 HEPES, 5 EGTA, 2.5 Na_2_ATP, pH 7.3 (with KOH), 290–295 mOsm/kg (Wilke et al., 2014).

### Substances

Tetraethylammonium (TEA, Sigma), NMDG (Sigma), 4-aminopyridine (≥99%; Sigma and Tocris Bioscience, Bristol, United Kingdom), 2-aminopyridine (2-AP; Sigma), 3-aminopyridine (3-AP; Sigma), 3,4-diaminopyridine (3,4-DAP; Sigma), 1,3-Bis-(2-hydroxy-5-trifluoromethyl-phenyl)-urea (NS 1643, Tocris), 10,10-*bis*(4-Pyridinylmethyl)-9(10*H*)-anthracenone dihydrochloride (XE991, Tocris), and *N*-[4-[[1- [2-(6-Methyl-2-pyridinyl)ethyl]-4-piperidinyl]carbonyl]phenyl] methanesulfonamide dihydrochloride (E-4031, Tocris) were diluted in extracellular solution to concentrations indicated in “Results.” All substances were applied locally via a glass capillary through a custom-made application system.

### Note on 4-AP Solutions

4-AP did not significantly change the pH of the extracellular solution at concentrations below 3 mM. After dilution of the substance, pH of the extracellular solution containing 5 mM or 10 mM 4-AP typically was about 8.1 or 9.0, respectively. As indicated in “Results,” in some experiments the pH of solutions containing 5 and 10 mM 4-AP was adjusted to 7.4 after dilution of 4-AP. At the concentration applied in the present study (3 mM), isomeric aminopyridines did not change pH of the extracellular solution (c.f. **Figure 4**).

### Data Analysis

Patch clamp recordings were analyzed with PatchMaster (HEKA) and IgorPro (Wavemetrics, Lake Oswego, OR, United States). Voltage dependence of activation was derived from tail current amplitudes using voltage protocols indicated: Tail currents were fitted with a two-state Boltzmann function with *I* = *I*_min_ + (*I*_max_ - *I*_min_)*/*(*1* + *exp*((*V* -*V*_h_)*/s*)), where *I* is current, *V* is the membrane voltage, *V*_h_ is the voltage at half maximal activation, and *s* describes the steepness of the curve. Results are shown as conductance-voltage curves, obtained by normalizing to (*I*_max_ -*I*_min_), obtained from fits to data of individual experiments. Time constants of deactivation were derived from double-exponential fits to deactivating current components at indicated potentials. For dose-response relations, current potentiation at 0 mV (normalized to baseline) was fitted to a Hill equation with *I*/*I*_b_ = *I*_b_ + (*I*_max_-*I*_b_)/(1 + (EC_50_/[S])^n_H_^), where *I* is the (normalized) current, *I*_b_ and *I*_max_ denote minimal and maximal currents at low and high drug concentrations, EC_50_ is the concentration at the half maximal effect, [S] is the drug concentration and *n*_H_ is the Hill coefficient (Leitner et al., 2012).

### Statistical Analysis

Isolated cells under investigation were randomly assigned to different treatment groups. Data recordings and analysis for experiments presented were not performed in a blinded manner. For some experiments, single recordings were normalized to base line values individually to account for baseline variations between cells. Statistical analysis was performed using two-tailed Student’s *t*-test/Wilcoxon–Mann–Whitney test, and when appropriate comparisons between multiple groups were performed with ANOVA followed by Dunnett test. Significance was assigned at *P* ≤ 0.05 (^∗^*P* ≤ 0.05, ^∗∗^*P* ≤ 0.01, ^∗∗∗^*P* ≤ 0.001). Data subjected to statistical analysis have *n* over 5 per group and data are presented as mean ± SEM. In electrophysiological experiments, *n* represents the number of individual cells and accordingly the number of independent experiments (no pseudo-replication).

## Author Contributions

MD, SE, BW, and ML planned and performed the experiments and analyzed the data. ML conceived study, designed the experiments, and wrote the paper together with MD. All authors revised and approved the final version of the manuscript. All experiments were conducted at the Institute of Physiology and Pathophysiology at the Philipps-University Marburg (Germany).

## Conflict of Interest Statement

The authors declare that the research was conducted in the absence of any commercial or financial relationships that could be construed as a potential conflict of interest.
