# Supplementary material for: Inverse Modulation of Neuronal Kv12.1 and Kv11.1 Channels by 4-Aminopyridine and NS1643
Source: Front Mol Neurosci. 2018 Jan 30;11:11. doi: 10.3389/fnmol.2018.00011 (PMC5797642; doi:10.3389/fnmol.2018.00011)
Supplement: Supplementary file 1 [file Data_Sheet_1.docx]

Supplementary Material
Inverse modulation of neuronal Kv12.1 and Kv11.1 channels by 
4-aminopyridine and NS1643
Marlen Dierich, Saskia Evers, Bettina U. Wilke, and *Michael G. Leitner
*Correspondence: Dr. Michael G. Leitner: leitnerm@staff.uni-marburg.de


Supplementary Figure 1. Measuring activity of human Kv12.1 channels in CHO cells.
Supplementary Figure 1. Measuring activity of human Kv12.1 channels in CHO cells.
(A+C) Representative recordings of human Kv12.1 channels transiently expressed in CHO cells measured with the following voltage protocols (c.f. Figure 1G): a 200 ms conditioning potential step to -120 mV (blue), -60 mV (black), 0 mV (red), or +40 mV (orange) was followed by 600 ms activating pulses at potentials from -140 mV to +10 mV (+10 mV increments). Tail currents were elicited at potentials that corresponded to conditioning pulses (-120 mV or 0 mV). Current traces for conditioning potential of -60 mV are also as shown in Figure 1A. (B) Summary of steady-state outward currents obtained from recordings as presented in (A). Amplitudes of outward currents were the same for all protocols indicating that channels were comparably activated with all voltage protocols.
(D) Slope factors derived from Boltzmann fits to the recordings changed with conditioning potentials. Slope factors were derived from recordings as shown here in (A+C) and in Figure 1G-I.
(E+F) Mode shift of human Kv12.1 channels was sensitive to the employed voltage protocol: In these experiments, tail currents were measured at hyperpolarized potentials (-120 mV) after 600 ms activating pulses from -140 mV to +20 mV (10 mV increments) and 200 ms conditioning potentials of -60 mV or 0 mV. (E) shows representative recordings of human Kv12.1 channels measured with these protocols and (F) shows the summary of voltage dependence of human Kv12.1 channels (solid line represents a Boltzmann fit to averaged data). Depolarized conditioning potentials of 0 mV induced a large shift of voltage dependence to hyperpolarized potentials also in these experiments. In these experiments, Vh was -29.8 ±1.3 mV and -61.8 ±1.1 mV after conditioning pulses of -60 mV and 0 mV, respectively (n=4, data derived from fits shown in (F) and recordings shown in (E)).


Supplementary Figure 2. Kv12.1 channels are not sensitive to E-4031, XE991, and TEA.
(A, D, G) Representative recordings of CHO cells transiently transfected with (A) Kv11.1, 
(D) Kv7.4 and (G) Kv7.2 before (black) and after (red) application of (A) E-4031, (D) XE991 and
(G) TEA (voltage protocols as indicated). These channels are well established targets of the respective K+ channel inhibitor. (B) Effect of E-4031 at a concentration generally applied to inhibit native Kv11 channels (20 µM) on steady-state outward currents mediated by Kv11.1 (left) and Kv12.1 (right).
(C) Kv12.1 channels were insensitive to E-4031 applied at concentrations between 1 µM and 100 µM.
(E) Averaged steady-state outward currents through Kv7.4 (left) and Kv12.1 (right) channels before and after application of 10 µM XE991, a concentration typically used to inhibit native Kv7 channels.
(F) Kv12.1-mediated currents were not affected by XE991 at concentrations between 1 µM and 100 µM. (H) Steady-state outward currents mediated by Kv7.2 (left) and Kv12.1 (right) before and after application of TEA (5 mM). In contrast to Kv7.2, Kv12.1 channels were insensitive to TEA at this concentration. (I) Dose-response experiments revealed that Kv12.1 channels were slightly inhibited by high TEA concentrations. When we applied 50 mM or 100 mM TEA, Kv12.1 channels were inhibited by approximately 10% and 17% (NaCl was substituted with TEA-Cl in these recordings).


Supplementary Figure 3. Inhibition of recombinant Kv11.1 channels by 4-AP. 
(A) Representative recordings of currents through Kv11.1 channels before (black) and after application of 3 mM 4-AP (green, upper panel) or 10 mM 4-AP (yellow; lower panel). Voltage protocol was as indicated. (B) Averaged time course of tail current amplitudes upon application of increasing concentrations of 4-AP (concentrations as indicated). (C) 4-AP inhibited Kv11.1 channels in a dose-dependent manner with an IC50 of about 2.6 mM and a Hill coefficient of about 0.7, in line with a previous report (Ridley et al., 2003). Parameters were derived from fits of averaged data to a Hill equation described in Methods (solid line represents this fit) (pH adjusted). 

Reference
Ridley, J.M., Milnes, J.T., Zhang, Y.H., Witchel, H.J., and Hancox, J.C. (2003). Inhibition of HERG K+ current and prolongation of the guinea-pig ventricular action potential by 4-aminopyridine. J Physiol 549, 667-672.


Supplementary Figure 4. Slow and incomplete inhibition of Kv12.1 channels by 10 µM NS1643. 
(A) Representative recordings from CHO cell transiently transfected with Kv12.1 before (black) and after (red) extracellular application of 10 µM NS1643 (Voltage protocol as indicated).
(B) Averaged time course of Kv12.1-mediated currents upon application of 10 µM NS1643 (currents measured at -20 mV in recordings as presented in (A)). Note that 10 µM NS1643 did not completely inhibit Kv12.1 currents and that time course of inhibition was much slower than inhibition induced by 30 µM NS1643 (c.f. Figure 5B in main text). 


Supplementary Figure 5. CHO cells transfected with plasmids encoding five different K+ channel subunits express large Kir2.1- and Kv7-mediated currents. 
In these experiments, CHO cells were transfected with equal amounts of plasmid DNA encoding Kv7.2, Kv7.3, Kv11.1, Kv12.1 and Kir2.1. This figure shows analysis of Kir2.1- and Kv7-mediated current components in these cells. For identification of Kv11.1 and Kv12.1, currents see Figure 6 in the main text. (A) Representative recordings of K+ currents in CHO cells transfected with the mix of K+ channel subunits before (black; left) and after (red; middle) application of the Kv7-specific antagonist XE991. XE991-sensitive currents (blue; right) were calculated by subtracting currents after XE991 from control currents (voltage protocol as indicated). (B-D) Analysis of steady-state currents. (B) Summary of steady-state currents elicited by voltage steps between -140 mV and +20 mV before and after application of XE991 (20 µM). CHO cells transfected with the mix of K+ channels showed large inward and outward currents at hyperpolarized and depolarized potentials, respectively. Inward currents demonstrated expression of functional Kir2.1 channels, and application of XE991 (20 µM) selectively inhibited outward currents demonstrating functional expression of Kv7 channels in these cells. Steady-state current amplitudes were analyzed at the end of the activating pulse from recordings as shown in (A). (C) Summary of steady-state inward currents elicited at -140 mV and outward currents at 0 mV before and after application of XE991 (20 µM). (D) Summary of XE991-sensitive currents at -140 mV and 0 mV (derived from recordings as shown in (A)).
